# Tumor and Peripheral Immune Status in Soft Tissue Sarcoma: Implications for Immunotherapy

**DOI:** 10.3390/cancers13153885

**Published:** 2021-08-01

**Authors:** Luana Madalena Sousa, Jani Sofia Almeida, Tânia Fortes-Andrade, Manuel Santos-Rosa, Paulo Freitas-Tavares, José Manuel Casanova, Paulo Rodrigues-Santos

**Affiliations:** 1 Laboratory of Immunology and Oncology, Center for Neuroscience and Cell Biology (CNC), University of Coimbra, 3004-504 Coimbra, Portugal; luana.sousa@student.uc.pt (L.M.S.); tania.andrade@student.fmed.uc.pt (T.F.-A.); 2 Life Sciences Department, Faculty of Sciences and Technology (FCTUC), University of Coimbra, 3000-456 Coimbra, Portugal; 3 Institute of Immunology, Faculty of Medicine (FMUC), University of Coimbra, 3004-504 Coimbra, Portugal; jani.almeida@student.uc.pt (J.S.A.); msrosa@fmed.uc.pt (M.S.-R.); 4 Center of Investigation in Environment, Genetics and Oncobiology (CIMAGO), Faculty of Medicine, University of Coimbra, 3000-548 Coimbra, Portugal; jmcasanova@fmed.uc.pt; 5 Coimbra Institute for Clinical and Biomedical Research (iCBR), Faculty of Medicine, University of Coimbra, 3000-548 Coimbra, Portugal; 6 Center for Innovation in Biomedicine and Biotechnology (CIBB), University of Coimbra, 3000-548 Coimbra, Portugal; 7 Clinical Academic Centre of Coimbra (CACC), 3000-075 Coimbra, Portugal; pftavares@chuc.min-saude.pt; 8 Coimbra Hospital and University Center (CHUC), Tumor Unit of the Locomotor Apparatus (UTAL), University Clinic of Orthopedics, Orthopedics Service, 3000-075 Coimbra, Portugal

**Keywords:** soft tissue sarcoma, immune monitoring, immunophenotyping, cytokines, immune checkpoints, gene expression

## Abstract

**Simple Summary:**

Soft Tissue Sarcomas are a rare and heterogeneous group of tumors, which have a characteristic complexity, leading to a difficult diagnosis and a lack of response to treatment. The aim of this review is to summarize the role of immune cells, soluble plasmatic factors, immune checkpoints; and the expression of immune-related genes predicting survival, response to therapy, and potential immunotherapeutic agents or targets in Soft Tissue Sarcomas.

**Abstract:**

Soft Tissue Sarcomas (STS) are a heterogeneous and rare group of tumors. Immune cells, soluble factors, and immune checkpoints are key elements of the complex tumor microenvironment. Monitoring these elements could be used to predict the outcome of the disease, the response to therapy, and lead to the development of new immunotherapeutic approaches. Tumor-infiltrating B cells, Natural Killer (NK) cells, tumor-associated neutrophils (TANs), and dendritic cells (DCs) were associated with a better outcome. On the contrary, tumor-associated macrophages (TAMs) were correlated with a poor outcome. The evaluation of peripheral blood immunological status in STS could also be important and is still underexplored. The increased lymphocyte-to-monocyte ratio (LMR) and neutrophil-to-lymphocyte ratio (NLR), higher levels of monocytic myeloid-derived suppressor cells (M-MDSCs), and Tim-3 positive CD8 T cells appear to be negative prognostic markers. Meanwhile, NKG2D-positive CD8 T cells were correlated with a better outcome. Some soluble factors, such as cytokines, chemokines, growth factors, and immune checkpoints were associated with the prognosis. Similarly, the expression of immune-related genes in STS was also reviewed. Despite these efforts, only very little is known, and much research is still needed to clarify the role of the immune system in STS.

## 1. Soft Tissue Sarcoma

Soft Tissue Sarcomas (STS) are a heterogeneous group of diseases of mesenchymal origin. STS represent approximately 1% of solid tumors [1]. This group comprises over 50 different histologic subtypes that affect patients of all ages [2]. Although they can occur anywhere in the body, the most common anatomic sites are the extremities (60–70%) and the abdomen and retroperitoneum (20%) [3]. In addition to being highly heterogeneous in anatomical localization and histology, they are also heterogeneous in terms of molecular characteristics and prognosis [4].

STS diagnosis is mainly based on histological interpretations, including immunohistochemistry, cytogenetic, and molecular analysis [5]. However, due to their rarity and heterogeneity, the diagnosis is challenging and requires expert analysis [6]. Therefore, a consensus and reproducible diagnostic criteria are crucial. The WHO classification provides an organization by tumor type, considering morphologic, immunohistochemical, and genetic features [7,8]. This classification also stratifies STS according to clinical behavior into benign, intermediate locally aggressive, intermediate rarely metastasizing, and malignant [7,8].

The increased availability of genomic technologies has provided a better understanding of sarcoma biology. STS can be divided into two groups based on genetic profiles: STS associated with specific genetic alterations and STS with nonspecific and nonrecurrent genetic alterations [5]. The first group includes chromosomal translocations that produce chimeric fusion genes, often encoding aberrant transcription factors, oncogenic mutations, or recurrent gene amplifications. These alterations may be tumor-specific or shared by several histological tumors with different histomorphologies and behaviors. In contrast to the STS associated with specific genetic alterations, the second group tends to have complex karyotypes, such as changes in chromosome number, unbalanced translocations, genetic deletions, and amplifications [5]. Concerning etiology, even though the majority is unknown, there are some genetic predisposal syndromes, such as Li-Fraumeni syndrome, Von Recklinghausen disease, or RB1 tumor-suppressor gene mutations that can lead to STS. Environmental factors, such as ionization, radiation, and chemical exhibitors, may also promote these sarcomas [6].

For localized STS, surgical resection with or without radiotherapy is the standard treatment. Unfortunately, STS recurs frequently as a locally inoperable or metastatic disease. For a locally advanced or metastatic disease, the usual treatment is chemotherapy [9]. Single-agent anthracycline is the first-line therapy and, for the second-line treatment, trabectedin and eribulin have demonstrated efficacy for some subtypes of STS [4]. 

Despite the remarkable improvement in cancer diagnosis and treatment, many patients do not respond to therapy. This limited effectiveness of current strategies is often attributed to the complexity of the disease. That is, at least partly, supported by the complex microenvironment where the tumor is growing and defeating the immune system. 

There is a growing interest in studying the immunological status of STS patients. The tumor microenvironment (TME) includes different populations of non-tumor cells, such as endothelial, stromal, cancer-associated fibroblasts and adipocytes, and immune cells [9]. The study of tumor-infiltrating and peripheral immune cells and mediators of the immune response may help to reveal the mechanisms related to tumor immunity. Moreover, such a study could identify potential biomarkers that favor an accurate prognosis, effective therapy response monitoring, and a refined approach to treatment. Recently, a transcriptomic analysis of >10,000 patients identified four distinct TME subtypes conserved across 20 different cancers: immune-enriched, fibrotic (IE/F); immune-enriched, non-fibrotic (IE); fibrotic (F); and immune-depleted (D). This TME subtyping strongly correlated with survival in most of the cancer types analyzed. The IE/F and IE TME were correlated with a better prognosis, while the F TME was linked to a worse prognosis. Furthermore, this study has also showed that patients with immune-favorable TME subtypes could benefit the most from immunotherapeutic approaches [10].

Concerning sarcomas, critical elements of peripheral blood and TME also play an essential role in predicting the response to therapy and are potential therapeutic agents or targets. Furthermore, a study from The Cancer Genome Atlas (TCGA) consortium proposed an association of the TME with prognosis in different STS histotypes [11]. Regarding the TME, the immune cells play an important role in controlling the progression of multiple tumor types. Nevertheless, in human STS, their characterization remains poorly defined. In a later study, Petitprez et al. developed a new classification and stratification of STS based on the composition of the immune microenvironment [12]. This classification was made up of five sarcoma immune classes with clearly different profiles and significantly different TME compositions. Each histological subtype was identified in each class, making it clear that the immune profile varies even between tumors with the same histology. This work also confirmed that the simplistic characterization of STS as “non-immunogenic” tumors does not apply to all, given that two sarcoma immune classes showed an elevated expression of genes specific to immune populations and the expression of immune-checkpoint-related genes. Furthermore, they also demonstrated that the immune microenvironment could be used to evaluate the prognosis and predict the response to immunotherapy.

The aim of this review was to summarize the prognostic and therapy response prediction value of immune cells, soluble plasmatic factors, immune checkpoints, and the expression of immune-related genes in STS patients, as well as their role in immunotherapeutic approaches.

## 2. The Role of Immune Cells in STS

### 2.1. Tumor-Associated Macrophages

Macrophages are vital innate immune cells present in tissues, and it has been suggested that they play a role in tumor development and progression [13]. They are differentiated by the local microenvironment into M1 or M2 macrophages, developing a pro- or anti-inflammatory response, respectively. Macrophages that are differentiated by the TME are called tumor-associated macrophages (TAMs). Due to several factors, for example, IL-4 and IL-13, an M2-like differentiation occurs in the TME, which facilitates tumor immune escape and metastasis [14,15]. M2-like TAMs block CD8 T cell-mediated anti-tumor immune response either directly, through their expression of inhibitor ligands, such as the programmed death-ligand 1 (PD-L1), or indirectly, via the C-C motif chemokine ligand 22 (CCL-22)-mediated recruitment of regulatory T cells (Tregs). A recent study detected, through immunohistochemistry, M2-like TAMs in all STS samples, while M1-like TAMs were only found in a few tumors and in a low density [16]. The presence of TAMs polarized toward a pro-tumoral phenotype in all the STS samples analyzed supports the possibility of targeting TAMs for STS treatment. TAMs could also be used to predict the clinical outcome. In several tumor types, this prognostic significance has already been shown [17,18]. However, concerning sarcomas, little is currently known. Still, the high density of M2-like macrophages, expressing CD163, and M1-like macrophages, identified by CD68 staining, were both significantly correlated with a poor outcome in non-gynecologic leiomyosarcomas [19]. Later, Kostine et al. also evaluated M2 and M1-like macrophages, and only the M2 phenotype was associated with worse survival rates for leiomyosarcoma [20]. Similarly, in myxoid liposarcoma (MLS), high levels of TAMs were also associated with poor survival [21]. More recently, a study performed with different types of STS identified TAMs as a poor prognostic for local recurrence, confirming the negative prognostic value of TAMs [22].

### 2.2. Tumor-Associated Neutrophils

Neutrophils make up a substantial proportion of the immune infiltrate in cancer, and their role has long been a matter of controversy. Similar to TAMs, in mouse models, it has been demonstrated that tumor-associated neutrophils (TANs) can retain some functional plasticity and can acquire different phenotypes based on specific features of the TME. In a TGF-β-rich environment, neutrophils usually acquire an N2 phenotype associated with a pro-tumor activity. On the contrary, in the presence of IFN-β or inhibition of TGF-β, neutrophils switch to an N1 profile, which is usually associated with anti-tumor activity. Although the tumor-promoting effects of N2 TANs have been demonstrated, human TANs remain underexplored [23]. Ponzetta et al. have shown that mice with profound neutropenia presented an earlier tumor development compared with wild-type mice [24]. Moreover, the adoptive cell transfer of neutrophils into sarcoma-bearing mice restores tumor growth to the level of the control group. These results prove that TANs are essential to restrain sarcomagenesis. The same study also showed a correlation between the high density of TANs and a better outcome in undifferentiated pleomorphic sarcomas (UPS). However, this correlation was not observed in other STS subtypes, such as dedifferentiated liposarcoma, leiomyosarcoma, and myxofibrosarcoma.

### 2.3. Tumor-Infiltrating Lymphocytes

Tumor-infiltrating lymphocytes (TILs) are strong indicators of tumor immunogenicity. TILs have been described in various malignant tumors, including STS, and some studies support the influence of TILs on the progression of some tumors [25]. It was observed that most STS patients had low TIL infiltration. However, in STS, TILs have been only reported considering a few STS subtypes in limited sample size studies. For these reasons, although the presence of TILs and their impact on positive outcomes have been demonstrated in several sarcoma subtypes, these reports may not be representative of all STS [12,26].

#### 2.3.1. T Cells

To explore the level of T cell infiltration in STS, two studies analyzed the expression profile of CD3E. The former suggested that T-cell infiltration could depend on the STS subtype and proposed that a highly mutated tumor type may have greater immunogenicity and a robust T-cell infiltrate [27]. In the latter, CD3E was highly expressed in some STS samples, such as rhabdomyosarcoma and alveolar soft part sarcoma, corroborating the idea that T-cell infiltration depends on the STS subtype [28].

CD8 T cells can mediate the lysis of neoplastic cells. For that reason, these cells are usually associated with a direct anti-tumor immune response. Furthermore, there is an influence of these cells on the clinical course of several types of tumors. However, the excessive and constant exposure of CD8 T cells to cancer antigens and inflammatory signals leads to a progressive loss of the T cell effector function; this is called “exhaustion”. Exhausted T cells can be characterized by the presence of inhibitory receptors; PD-1 and LAG3 are among them [29]. The analysis of CD8 T cells in the TME, including their receptor repertoire, has been increasing, given the availability of new activating drugs [30]. 

CD4 T cells are also required for anti-tumor immunity. They comprise diverse subsets with different and sometimes opposing roles in TME, upregulating or downregulating the immune response. Regarding their anti-tumor activity, they are responsible for enhancing the cytotoxic function of CD8 T cells, increasing clonal expansion, functioning as antigen-presenting cells, for example [31,32]. Fresh tumors resected at surgery and analyzed by flow cytometry have shown a greater prevalence of CD4 than CD8 T cells in well differentiated and dedifferentiated retroperitoneal liposarcoma [30]. The majority of tumor-infiltrating lymphocytes were CD4 ‘helper’ T cells, and most CD8 T cells expressed their programmed cell death protein 1 (PD-1). This information suggests that CD8 T cells have been triggered by tumor antigen but are suppressed.

On the contrary, D’Angelo et al. described a greater prevalence of CD8 than CD4 T cells in STS tumors [33]. Those tumors were more likely to express PD-L1 and PD-1, once more suggesting the inactivation of these cells. Another study analyzed the density of T cells in 28 tumors diagnosed as undifferentiated sarcoma [34]. They observed a positive correlation between the density of CD8 T cells and the density of macrophages. Since some studies have indicated that TAMs suppress the cytotoxic functions and chemotaxis of CD8 T cells in other tumors, it would be interesting to know whether TAMs also affect CD8 T cells in undifferentiated sarcomas [35,36].

**Figure 1:**
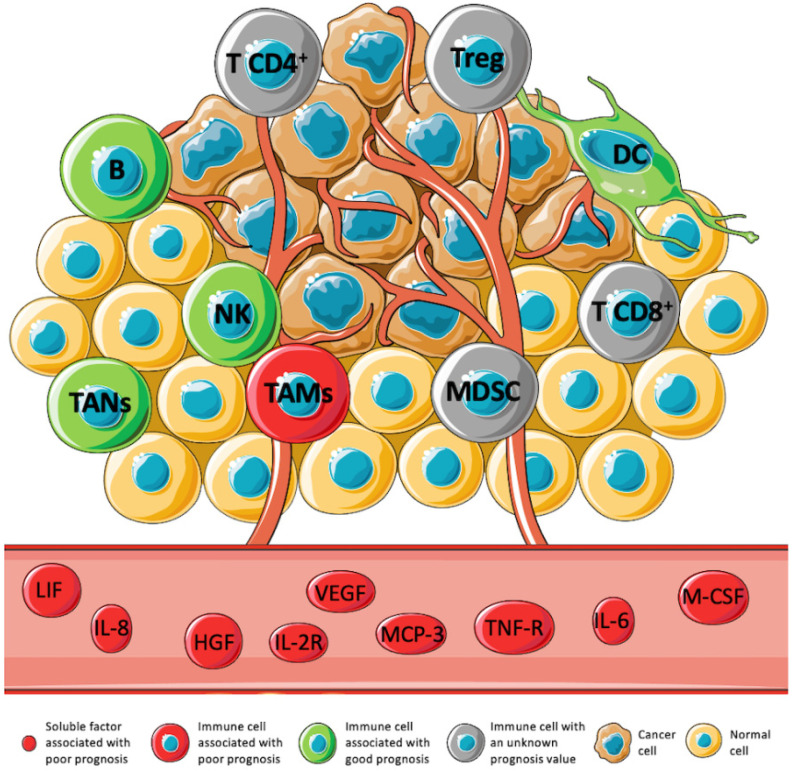
Expression levels of immune cell subtypes, cytokines, chemokines, growth factors, and soluble receptors and their prognostic value in STS. The TME has been associated with the prognosis in several tumors. However, in STS, this association is still underexplored. Immune cells such as B cell, DC, TANs, and NK have been associated with a positive prognosis (green). On the contrary, TAMs, and some soluble factors: LIF, IL-8, HGF, IL-2R, VEGF, MCP-3, TNF-R, IL-6, and M-CSF, have been associated with a negative prognosis (red). The prognostic value of MDSCs, Tregs, CD4 T cells, and CD8 T cells is not clear yet (gray) [11,22,24,26,40,42,44,45,46,47].

Several studies have been trying to correlate the frequency of immune cells with the prognosis in STS (Figure 1). An association between CD8 T cells and improved outcomes has been observed [26,37,38]. However, conflicting studies have also observed an association with poor outcomes [39]. Moreover, there are also other studies that state that there is no statistical significance in this correlation [34,40]. Concerning CD4 T cells, the controversy remains. Although in some studies, CD4 T cells have been associated with a positive outcome [40,41], the opposite, an association with a poor prognosis, has also been observed [33,39]. In addition, some studies do not observe any significant prognostic value [38]. These discrepancies between studies may be due to the differences in methodology, antibody clones, and cutoff values used [39]. Furthermore, studies have indicated that these cell frequencies vary between STS subtypes and treatments [42,43]. For these reasons, the differences in sarcoma subtypes and the limited size of patient cohorts may also explain the discrepancies in the results.

#### 2.3.2. B Cells

Recent data have shown that B cells can shape immune responses in tumors [48]. However, the association of these cells with disease prognosis has been a reason for disagreement. In several tumors, it described an association with a good prognosis. However, the opposite has been reported, too [16]. In well-differentiated and dedifferentiated retroperitoneal liposarcoma, B cells were found, generally with a low frequency, in some of the tumors analyzed [30]. In 2011, it was suggested that B cells could be an independent favorable prognostic factor in STS patients with wide resection margins [40]. Later, the association of B cells with a good prognosis was supported by Tsagozis et al. [16]. This study also observed an absence of B cells in many tumor areas, corroborating previous works. 

Recently, Petitprez et al. published an integrative analysis dedicated to B cells and their influence on sarcoma survival and immunotherapy response [12]. They found that B cells are a key discriminative feature of a group of patients with improved survival and a better response to PD-1 blockade therapy, confirming their role as a positive prognostic factor. In addition, Helmink et al. found that B cell markers were the most differentially expressed genes in the tumors of STS responsive patients versus tumors of patients that did not respond to immunotherapy [49]. This data confirmed once more the potential of B cells as biomarkers.

#### 2.3.3. Natural Killer Cells

Natural killer cells (NK) have the ability to lyse transformed cells [50]. Therefore, these cells play an important role in cancer immunosurveillance [51]. Studies of other tumors, such as clear cell renal cancer and non-small cell lung cancer, have evaluated the role of NK cells in the TME and the relationship between the infiltration of NK cells and the clinical outcome [52,53,54]. 

There have been a few studies of the NK cell function in STS. One of them used flow cytometry to detect infiltrating NK cells, generally in a low density, in some well-differentiated and dedifferentiated retroperitoneal liposarcoma tissues [30]. Another study analyzed the tumor immune microenvironment signatures of 206 STS patients [11]. Regarding NK cell infiltrate, they reported that these immune cells were the only cells to correlate significantly with better disease-specific survival (DSS) in several sarcoma types. Later, Judge et al. also correlated tumor-infiltrating NK cells with improved survival in STS [26]. 

Although NK cells display an even higher cytolytic activity compared to CD8 T cells, their cytolytic function may be drastically dependent on the balance of activating and inhibiting surface receptors [55]. One activating receptor, NKp30, was found to be particularly downregulated in peripheral and tumor-infiltrating NK cells in gastrointestinal sarcoma (GIST) when compared to the circulating NKp30+ NK cells of healthy volunteers [56]. Nevertheless, the levels of total NK cells were similar in GIST and healthy volunteers. These results highlight the importance of further studies focused on NK cell receptors, since they affect the functions of these cells without affecting their frequency.

### 2.4. Dendritic Cells

Dendritic cells (DCs) also play an essential role in the immunological environment. The TCGA analyzed the immune cell infiltrates based on tumor gene expression signatures and showed a correlation between the presence of tumor-infiltrating DCs and improved DSS in UPS and myxofibrosarcoma [11]. Although there is a lack of studies concerning DCs in STS, this conclusion suggests an important role of antigen presentation in immune responses against these tumors.

### 2.5. Suppressor Cells

#### 2.5.1. Regulatory T Cells

Regulatory T cells (Tregs) are physiologically suppressive cells and play an important role in maintaining the homeostasis of the immune response. They can produce immunosuppressive cytokines such as interleukin 10 (IL-10) and tumor growth factor-β (TGF-β), they can express negative costimulatory molecules such as cytotoxic T-lymphocyte-associated protein 4 (CTLA-4), PD-1, or PD-L1, and they consume cytokine interleukin 2 (IL-2). These functions lead to an inhibition of T lymphocytes and the promotion of immune escape [57]. Studies of other tumors have associated high density of tumor-infiltrating Tregs with a poor outcome. However, the opposite has also been demonstrated [58]. In STS, D’Angelo et al., using immunohistochemistry, observed a high density of tumor-infiltrating Tregs in 75% of STS patients, most of them of GIST histology [33]. Later, another study evaluated tumor-infiltrating Tregs by immunohistochemistry and showed an association between the increased infiltration of these cells and a poor prognosis in STS [44]. However, an association has also been found between a greater percentage of Tregs, analyzed by multiplex immunofluorescence, and a better outcome [59]. The same study also correlated the increased tumor-infiltrating Tregs with a better response to pembrolizumab, anti-PD-1 monotherapy. Despite this, it has also been suggested that Tregs are not associated with STS prognosis [26,38]. Due to these controversial results and the limited number of studies, the prognostic significance of Tregs remains undefined.

#### 2.5.2. Myeloid-Derived Suppressor Cells

Myeloid-derived suppressor cells (MDSCs) are another subset of suppressive cells that can facilitate tumor immune escape, impairing the function of T cells, NK cells, and DCs. These immature myeloid cells can be phenotypically divided into early-MDSCs (e-MDSCs), monocytic MDSCs (M-MDSCs), and polymorphonuclear MDSCs (PMN-MDSCs) [60,61].

A study performed by Highfill et al. sought to investigate whether there was an expansion of MDSCs in rhabdomyosarcoma, the most common soft tissue sarcoma of childhood [60]. They used mice bearing rhabdomyosarcoma and observed, by flow cytometry, an expansion of MDSCs, preferentially PMN-MDSCs, localized at the tumor site. It was demonstrated that PMN-MDSCs have an essential role in rhabdomyosarcoma immune escape. Preventing the trafficking of these cells to the tumor could also improve the efficacy of checkpoint blockade. The role of MDSCs in human STS tumors remains underexplored.

## 3. Soluble Factors: Cytokines, Chemokines, Growth Factors, and Others

The network of pro- and anti-inflammatory cytokines and chemokines orchestrates the immune cell signaling and function and, as such, largely contributes to the complexity of the TME. Cytokines have been studied in a broad range of tumors, and their involvement in cancer development, progression, and recurrence has been suggested. Moreover, the cytokine profile might be a prognostic factor for clinical outcome [62,63]. The prognostic value of cytokines, chemokines, growth factors, and soluble receptors in STS is summarized in Figure 1. 

As well as cytokines, chemokines have multifaceted roles in tumor development and progression, promoting malignancy or restricting tumor growth [64]. Likewise, growth factors and soluble receptors also play a significant role in TME [65,66].

Preliminary studies have found an elevated serum level of some cytokines, growth factors, and immune-related soluble receptors in patients with STS. Higher serum levels of vascular endothelial growth factor (VEGF) and fibroblast growth factor (FGF) have been reported. They promote angiogenesis, facilitating the tumor’s growth and increased metastatic spread. Furthermore, VEGF also promotes the proliferation of immunosuppressive cells and T cell exhaustion, contributing largely to immune escape and cancer development [67,68,69]. In addition, increased serum levels of interleukin 6 (IL-6), receptors for TNF (TNF-RI and TNF-RII), interleukin 2 receptor α (IL-2Rα), interleukin 10 (IL-10), macrophage-colony stimulating factor (M-CSF), and interleukin-8 (IL-8) were also found in STS patients [45,70,71].

Rutkowski et al. analyzed the serum levels of 13 cytokines and soluble receptors in STS patients before treatment [45]. The results confirmed the elevated levels of VEGF, FGF, IL-6, TNF RI, TNF RII, IL-2Rα, IL-10, M-CSF, and IL-8 stated above. Furthermore, they tried to correlate the serum levels of these cytokines with clinic-pathological features. IL-2Rα, TNF RI, M-CSF, and VEGF correlated with tumor size, IL-8 was associated with tumor grade, and IL-6 appeared to be correlated with tumor size, grade, and metastases. Additionally, it was proved that IL-6 and IL-8 were correlated with decreased survival [45]. 

In relation to IL-6, a few more studies have confirmed the association of its serum levels with survival. Hagi et al. observed high levels of IL-6 associated with the presence of STS and proposed that IL-6 could be used as a marker for the differential diagnosis [72]. Furthermore, they confirmed the correlation between elevated IL-6 serum levels and decreased survival [69]. 

Wysoczynski et al. proposed that leukemia inhibitory factor (LIF) promotes the progression and the metastatic behavior of rhabdomyosarcoma cells, contributing to the resistance of rhabdomyosarcoma to conventional treatment [46]. Later, Wysoczynski found that IL-8 was a pivotal pro-angiogenic factor in rhabdomyosarcoma cells during hypoxia [73]. Still, in rhabdomyosarcoma cells, another study showed that tumor cell progression seemed to be regulated by the interleukin-4 receptor (IL-4R)-dependent signaling pathway, highlighting the role of IL-4 in this common type of STS [74]. 

TNF was also found in high levels in STS patient serum [45]. Similar to IL-6, the correlation between TNF and tumor grade, size, metastases, or recurrence was investigated. However, there was no significant association between the serum levels of TNF and these clinic-pathological features. Similarly, no association between these features and serum levels of IL-10 and granulocyte colony-stimulating factor (G-CSF) was demonstrated in STS patients [45]. 

Regarding IL-2Rα, its higher level in STS patients has been correlated with tumor size. Another study performed in 2012 suggested that a low serum level of IL-2Rα was associated with prolonged overall survival (OS) [47]. In this same study, Sleijfer et al. also indicated that low monocyte chemotactic protein-3 (MCP3) and hepatocyte growth factor (HGF) levels were associated with extended progression-free survival (PFS). However, they mentioned that these associations might be false-positive ones, so these results should be interpreted with caution and confirmed by more studies.

## 4. Expression of Immune Checkpoints and Their Ligands in STS

Immune checkpoints are essential in regulating the immune response. In cancer, they can be dysregulated, working as an immune resistance mechanism [75]. 

**Figure 2:**
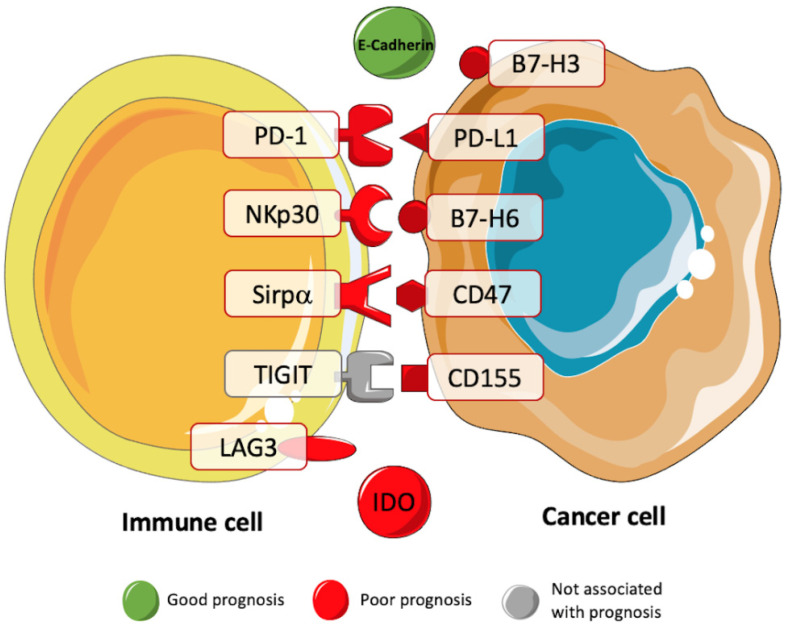
Prognostic value of immune checkpoints in STS. Several studies have been trying to correlate the presence of immune checkpoints with the prognosis of patients with STS. These studies have showed a negative prognostic value for B7-H3, PD-1, PD-L1, NKp30, B7-H6, Sirpα, CD47, CD155, LAG3, and IDO (red). A positive prognostic value was associated with the immune checkpoint E-Cadherin (green) [15,26,39,76,77,78,79,89,90,91,92].

In 2013, the impact of the immune checkpoints PD-1 and PD-L1 in STS (Figure 2) was evaluated for the first time [76]. The result from immunohistochemistry showed an intratumoral infiltration of PD-1 positive lymphocytes and the expression of PD-L1 in most STS samples. Additionally, PD-1 positivity, PD-L1 positivity, and the combined PD-1/PD-L1 pattern were independent prognostic indicators of OS and event-free survival. Furthermore, more studies have evaluated these immune checkpoints, the majority by immunohistochemistry, and confirmed the presence of PD-1 and PD-L1, and their association with a negative prognosis [77,78,79,80,81,82,83,84,85,86]. However, in some studies, PD-1 and PD-L1 expression appear to be low or absent, and the PD-L1 expression has not been associated with the outcome in STS [26,33,42,59,77,81,87]. Wunder et al. showed recently that the PD-1 and PD-L1 expression depended on the STS subtype and the prognostic value of PD-L1, justifying the discrepancies between studies with different subtypes of STS [88]. In addition, these discrepancies may also be due to the use of different methods of expression assessment, cutoff values, antibody clones, and tissue samples analyzed before and after therapeutical interventions [37,88].

PD-1 and PD-L1 expression levels have also been correlated in some studies with T-cell infiltration, and PD-L1 expression has been associated with more PD-1 positive TILs [27,79].

Although the presence and prognostic value of these immune checkpoints has been controversial and underexplored in this type of tumor, they might still have a role in predicting the prognosis of STS patients. Furthermore, the expression of these immune checkpoints may also indicate the patients who will benefit from PD-1 therapies. In 2020, a study concluded that STS patients who responded to pembrolizumab, an anti-PD-1 monotherapy, exhibited more PD-L1-expressing macrophages than non-responders [59].

Other immune checkpoints have been studied in several tumors, but there are only a few reports for STS. A recent study analyzed the expression of the B- and T-lymphocyte attenuator (BTLA) in sarcoma and found a lower expression mainly in CD4 TIL [77]. The same study also showed a high expression of lymphocyte-activation gene 3 (LAG3) on CD8 TILs. Other studies analyzed the expression of LAG3 by immunohistochemistry [39]. They confirmed its overexpression on TILs and found a significant association of LAG3 expression with a poor clinical outcome. Ishihara et al. suggested that a lower expression of indoleamine-pyrrole 2,3-dioxygenase 1 (IDO-1) was associated with a better prognosis in UPS [89]. E-Cadherin has also been studied in STS. It has been suggested that E-Cadherin has a possible role in the maintenance of epithelial architecture [93]. Furthermore, it was observed that upregulated E-Cadherin expression was associated with a better prognosis in STS patients [90,94]. The expression of B7-H6 and B7-H3 has also been evaluated in metastatic gastrointestinal stromal tumors and rhabdomyosarcoma, respectively [91,92]. In both studies, the expression of these molecules was associated with a worse prognosis. Dancsok et al. evaluated the immune checkpoints CD47 and Sirpα expression in sarcomas for the first time [15]. Through immunohistochemistry, the expression of both macrophage-related immune checkpoints was correlated with an adverse prognostic factor. Recently, the expression of the exhaustion marker T cell immunoreceptor with Ig and ITIM domains (TIGIT) was assessed in STS samples [26]. Although TIGIT expression was not associated with survival, the expression of its dominant ligand CD155 was associated with worse OS using the TCGA. 

## 5. Immune-Related Gene Expression in STS

**Figure 3:**
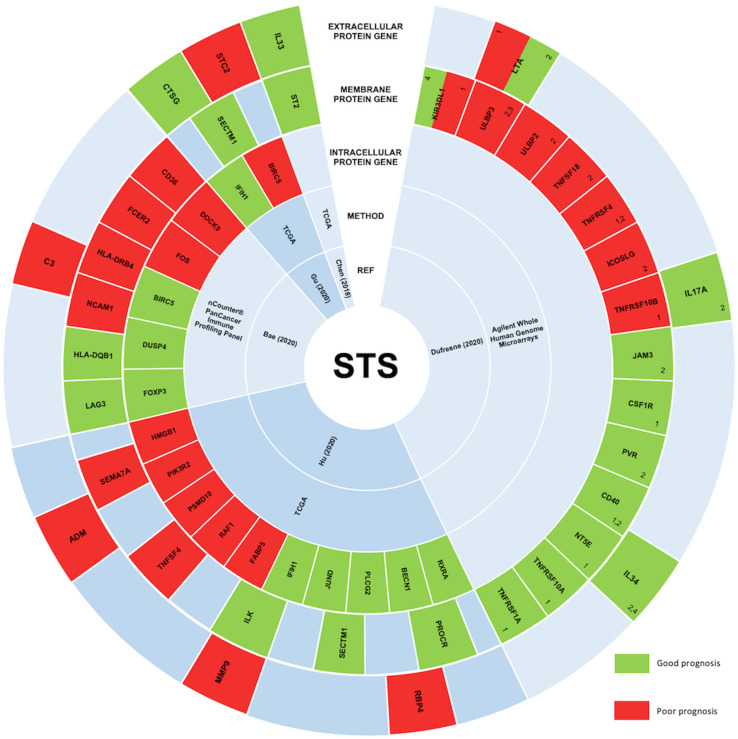
STS studies correlating the expression levels of immune-related genes and their prognostic significance. In STS, the expression of immune-related genes remains underexplored, and consequently, the prognostic value of these genes is still unclear. However, five main studies aimed at understanding this correlation, and their results are represented in this figure. Immune-related genes correlated with a good prognosis in STS are represented in green. On the other hand, immune-related genes associated with a bad prognosis are represented in red. From the peripheral to the center, circles represent genes encoding extracellular proteins, genes encoding transmembrane proteins, genes encoding intracellular proteins, the method used, and the respective study’s first author and publication year [99,100,101,102,103]. ^1^ Prognostic value in synovial sarcomas; ^2^ Prognostic value in gastrointestinal stromal tumors; ^3^ Prognostic value in myxoid liposarcomas; ^4^ Prognostic value in sarcomas with complex genetics.

Studies of lung cancer, ovarian cancer, head and neck squamous cell carcinoma, and renal cancer have suggested that immune-related genes (IRGs) may be used as prognostic biomarkers [95,96,97,98]. The IRG expression is underexplored in STS, and its prognostic significance remains unclear (Figure 3).

In STS, high and low transcription levels of IL33 and its receptor ST2 were associated with the recruitment of CD8 T cells and the recruitment of Tregs and MDSCs, respectively [99]. Moreover, in the same report, both IL33 and ST2 levels were associated with a better outcome.

Recently, the gene expression of 364 differentially expressed IRGs was analyzed [100]. It was established that 18 of these genes were significantly associated with overall OS or/and with PFS, validating their value as prognostic biomarkers. Likewise, Dufresne et al. analyzed the expression of 93 genes encoding for immune checkpoints and membrane proteins in 253 STS samples [101]. This analysis showed a correlation between the immune signature and each sarcoma subgroup, concluding that the prognostic value could depend on the group. Another study constructed an immune gene-related prognostic model using five immune-related prognostic genes: IFIH1, CTSG, STC2, SECTM1, and BIRC5 [102]. These five genes had an effective performance in risk stratification of patients, showing their potential as biomarkers for predicting the response of STS patients to immunotherapy. In addition, in 2020, the analysis of high-grade STS tissue samples, divided according to OS, identified seven genes (C3, CD36, DOCK9, FCER2, FOS, HLA-DRB4, and NCAM1) correlated with a poor prognosis, and six genes (BIRC5, DUSP4, FOXP3, HLA-DQA1, HLA-DQB1, and LAG3) correlated with a good prognosis [103].

## 6. Peripheral Blood Immune Status

The immunological status of peripheral blood in patients with STS remains unclear, just as its role as a prognostic indicator. 

The circulating monocyte count has been studied recently as a marker of poor prognosis in several tumors [104]. In addition, the correlation between the increasing monocytes and decreasing lymphocytes with tumor growth and progression has already been proved in cancer populations [104,105]. In 2014, the lymphocyte/monocyte ratio (LMR) was studied for the first time in STS patients [106]. They concluded that the pre-treatment LMR ratio could act as a negative prognostic factor. Jiang et al. also analyzed the monocyte ratio in 124 STS patients [107]. Their analysis observed a significant association between poor prognosis for OS and PFS, and the presence of a monocyte ratio > 1, which is in line with studies concerning other tumors. In addition to being a poor prognosis factor, a low LMR indicates systemic inflammation in cancer, including STS. However, the association between inflammation indexes and the prognosis has been challenging and controversial. A study performed in 2019 evaluated 26 cases of STS and did not find significant differences in OS and PFS associated with the LMR [108].

Two meta-analyses aimed at evaluating the effect of neutrophil-to-lymphocyte ratio (NLR) in STS [109,110]. Both concluded that higher NLR was associated with poor OS, disease-free survival (DFS), and PFS. Although multiple studies have proved an association between different cellular ratios with the prognosis for several tumors, data for STS are still sparse [106]. The peripheral immunological status of STS was investigated by Kim et al. in 2021; they observed that a high level of M-MDSCs was associated with poor DFS and PFS [111]. In the same way, high levels of T-cell immunoglobulin and mucin-domain containing-3 (Tim-3) positive CD8 T cells were associated with lower DFS. On the contrary, high levels of NKG2D positive CD8 T cells were significantly associated with longer DFS times. The collection of tumor samples is usually difficult; therefore, more studies based on a minimally invasive method, such as collecting peripheral blood, are needed.

The aim of another study was to analyze the immune cells in both peripheral blood and tumor tissue [26]. The data showed that NK and T cells are both more activated and exhausted in tumor tissue than in circulation when comparing these two locations. Concerning NK cells, both CD56^bright^ and CD56^dim^ subsets were found in peripheral blood. However, in tumor tissues, CD56^bright^, the less mature and cytotoxic subset, appears to be less prevalent. The activation marker CD69 was also evaluated, and it is more expressed in both NK subsets in the tumor, compared to the peripheral blood. Similarly, the expression of the receptor of NK and T cell exhaustion TIGIT was increased in the tumor. 

Regarding NK cells from peripheral blood, Bücklein et al. analyzed this cell subset in two groups of STS patients: chemotherapy-naïve STS patients and STS patients with a progression or relapse after chemotherapeutical treatment [112]. In both, NK cells were found to be dysfunctional during a chromium release assay using K562 cells as targets. The CD56^dim^ NK cell subset frequency, studied using flow cytometry, was significantly lower in the blood from STS patients with a progression or relapse after therapy when compared to healthy donors. These conclusions could be specific to STS patients, since these alterations were not found in NK cells from renal cell carcinoma patients. In addition, a decreased expression of NKG2D, CD3ζ, and perforin was found and associated with the activation of NK cells in the second group of patients. On the contrary, Delahaye et al. did not find significant differences in the levels of peripheral NK cells nor in the NKG2D expression in GIST patients when compared to healthy volunteers [56]. However, they showed that a predominant expression of the immunosuppressive NKp30c isoform of the NKp30 receptor was associated with an unfavorable outcome. 

## 7. Immunotherapy in STS

In 1891, William B. Coley injected streptococcal organisms into a patient with sarcoma. The injection stimulated the immune system, and the sarcoma disappeared. After this successful experiment, he treated hundreds of patients with sarcomas, including STS. Coley initiated the discipline of cancer immunotherapy and demonstrated the possible use of this type of therapy for this disease [113].

It is now clear that the immune microenvironment is highly variable in STS, and this variability is frequently justified by STS heterogenicity. Despite this heterogenicity, clinical trials continue to incorporate various sarcoma subtypes to obtain the minimum number of patients required. Although there have been hints of positive responses to immunotherapy trials for STS, most trials have been negative or are not representative of all STS subtypes. Currently (July 2021), there are 85 clinical trials focused on immunotherapy in STS. Phase II and phase III clinical trials that have been completed and targeting the immune system in STS are shown in Table 1.

As was mentioned before, the expression of PD-1 and PD-L1 were present in some studies and absent in others, which appears to depend on the STS subtype. The presence of these immune checkpoints in some subtypes offers a promise for immunotherapy based on checkpoint inhibitors in these specific subtypes. Unfortunately, clinical trials testing immune checkpoint inhibitors in STS have not showed the impressive results achieved for many other cancers. The intention of the first study was to analyze the efficacy of targeting the immune checkpoint CTLA-4 with ipilimumab in synovial sarcoma, but neither a clinical benefit nor immunological activity was demonstrated [114]. Similarly, uterine leiomyosarcoma patients did not respond to anti-PD-1 antibody nivolumab in a phase II study [115]. Later, the clinical trial SARC028 tested the anti-PD-1 therapy with pembrolizumab. Promising responses for specific subtypes were observed in this trial, such as UPS and dedifferentiated liposarcoma. Moreover, the response to pembrolizumab was correlated to higher tumor-infiltrating lymphocytes at the baseline. Based on these promising results for specific subtypes of STS and in specific immune microenvironments, further research and correlative studies are required to improve the selection of patients for future clinical trials with immune checkpoint blockade in STS.

Adoptive cell therapy is based on the manipulation, modulation, and selection of immune cells to eliminate the tumor, overcoming the immune system’s tolerance to cancer cells. As sarcomas appear to be one of the tumors most vulnerable to NK cell cytotoxicity, NK cell-based therapies seem to be a promising alternative treatment [116]. In 2010, it was demonstrated that rhabdomyosarcoma is sensitive to expanded NK cells [117], and phase I and II clinical trials of expanded haploidentical NK cells in rhabdomyosarcoma patients have begun (NCT02409576). The aim of another ongoing clinical trial is to combine cryosurgery and multiple NK immunotherapies (NCT02849366) (Table 1). Similar to NK cells, lymphocytes could also be harvested from the patient or a donor, expanded, and then reinfused into the patient. Although the use of TILs against STS is poorly investigated, two ongoing phase II clinical trials have started. One of them proposes a donor lymphocyte infusion in patients with relapsed malignancies, including sarcoma (NCT00003887). The other is trying to eradicate minimal residual disease in sarcomas, including alveolar rhabdomyosarcoma, with autologous T cell transplantation concomitant with the tumor-specific peptides vaccine (NCT00001566). Alternatively, genetically engineered T cells expressing receptors for specific recognition of the cancer testis antigen New York esophageal squamous cell carcinoma-1 (NY-ESO-1) could be a promising strategy, since the expression of NY-ESO-1 in some subtypes of STS has been demonstrated, especially in synovial sarcomas [118,119]. In this STS subtype, a T-cell receptor-based gene therapy against NY-ESO-1 demonstrated promising results [120]. In another pilot study, an autologous T-cell expressing T-cell receptor recognizing NY-ESO-1 confirmed previous results with an anti-tumor response in 50% of metastatic synovial sarcoma [121]. Considering all these previous promising results, the aim of an ongoing clinical trial is to create an immune response against NY-ESO-1 antigen with a CDX-1401 cancer vaccine (NCT00948961) (Table 1).

Cancer vaccines are a strategy to treat tumors. These vaccines attempt to elicit an immune response against tumor cells through the active manipulation of DCs. However, in addition to other limited reports of DC-based vaccination in STS, a study performed in 2017 indicated that the treatment is effective only in a small number of patients [122]. Several current clinical trials use vaccination with autologous dendritic cells to try to strengthen the immune system against sarcomas, including STS (NCT01347034; NCT02496520; NCT00365872). Peptide vaccination could also be an approach to treat STS, and clinical trials are testing peptide vaccines to enhance the immune response in STS (Table 1).

Clinical trials concerning immunotherapy for STS have, so far, shown limited and inconclusive results, which is largely due to the lack of representativity of several STS histologic types in the studies. However, attempts are still ongoing to identify biomarkers for monitoring immunotherapy and predict clinical outcome [123,124].

## 8. Future Perspectives

Beyond the necessity of large-scale studies on tumor-infiltrating immune cells and their role in clinical features, it is also necessary to pair the analysis of tumor samples with peripheral blood samples to understand whether the information obtained about the circulating immune cells could be used to predict disease outcome or the response to treatment. The collection of peripheral blood is a minimally invasive procedure, which facilitates sample harvesting and consequently increases the number of patients who could undergo such a process and would allow patient monitoring during the treatment.

Regarding the soluble factors, there is still much to be learned about the array of these factors secreted by the tumor and their activity and interactions in TME. Given the pleiotropic and redundant nature of the soluble factors, the therapeutical target should be the balance between pro- and anti-inflammatory ones instead of the inhibition or activation of one in particular.

Treatments targeting immune checkpoints may represent a promising approach for other types of cancers as well. Nevertheless, it is necessary to select the patients who will benefit from this type of therapy carefully. Regarding IRGs, there are still only very few studies, so more research is required to understand the potential functional mechanisms of IRGs and their role in STS. The dual role of immunity in cancer leads us to believe that combination approaches that both stimulate protective host responses and inhibit immune subversion tactics might be more efficacious. The heterogenicity of STS implies that a “one size fits all” approach may be less successful. Furthermore, comprehensive immune profiling in combination with the evaluation of clinical features will be important to predict the response to therapy and survival. Lastly, the immune profiling of each patient might lead to personalized therapy.

The knowledge accumulated regarding tumor and peripheral immune status could be helpful in designing novel immunotherapeutic approaches for STS.

## 9. Conclusions

STS have been treated as “non-immunogenic” tumors until now. However, this current work has proved that this characterization did not apply to all of them, since elements of the immune system were highly expressed in some STS samples. These elements, including immune cells, soluble plasmatic factors, immune checkpoints, and the expression of immune-related genes have been correlated with STS prognosis. Furthermore, their role in predicting the response to therapy and their potential as therapeutical agents or targets has been proven in STS. The infiltration of B cells, NK cells, TANs, and DC in STS tumors were correlated with a better outcome. On the contrary, TAMs were associated with a negative prognostic value. Regarding infiltrating CD8 T, CD4 T, and Tregs, their role in the outcome of the disease remains controversial. Some soluble plasmatic factors such as LIF, IL-8, HGF, IL-2Ra, VEGF, MCP-3, TNF-RI, IL-6, and M-CSF were associated with a negative prognosis in STS. Nevertheless, only a few studies have tried to understand their role in this type of cancer. A favorable prognostic value was associated with the immune checkpoint E-Cadherin, and a negative prognostic value was associated with the presence of B7-H3, PD-1, PD-L1, NKp30, B7-H6, Sirpα, CD47, CD155, LAG3, and IDO. Likewise, immune-related genes such as IL-33, ST2, BIRC5, DUSP4, FOXP3, HLA-DQA1, HLA-DQB1, and LAG3 were associated with a better outcome, while C3, CD36, DOCK9, FCER2, FOS, HLA-DRB4, and NCAM1 were correlated with a worse outcome. In another study, an immune gene-related prognostic model using IFIH1, CTSG, STC2, SECTM1, and BIRC5 showed potential to predict the response of STS patients to immunotherapy. The immunological status of peripheral blood in STS is still largely unknown. Increased LMR and NLR ratios have been associated with a poor prognosis in some studies. Higher levels of M-MDSCs and Tim-3 positive CD8 T cells also appear to be negative prognostic markers. On the contrary, NKG2D-positive CD8 T cells were correlated with a better outcome.

The main limitations that concern the studies mentioned above are the small sample sizes, the short follow-up, and the use of restricted STS histology types. Taking this into account, the studies might not be representative of the whole. In addition, in most of these studies, the stage of STS and treatments were not considered and might have a significant impact on prognosis. For these reasons, a large-scale prospective study, investigation of each subtype, and studies that consider the STS stage and treatment are warranted to substantiate and validate the results discussed in this article.

## Figures and Tables

**Table 1:** Completed phase II and III clinical trials for immunotherapy in soft tissue sarcomas.

	NCT Identifier	Phase	Enrollment	Title	Interventions
**Adoptive Cell therapy**	NCT02849366	I and II	30	Combination of Cryosurgery and NK Immunotherapy for Recurrent Sarcoma	Cryosurgery
					NK cell immunotherapy
	NCT00001566	II	42	A Pilot Study of Autologous T-Cell Transplantation With Vaccine Driven Expansion of Anti-Tumor Effectors After Cytoreductive Therapy in Metastatic Pediatric Sarcomas	Therapeutic autologous dendritic cells
					Indinavir sulfate
					Peripheral blood stem cell transplantation
	NCT00003887	II	Not	Lymphocyte Infusion in Treating Patients With Relapsed Cancer After Bone Marrow or Peripheral Stem Cell Transplantation	Peripheral blood lymphocyte therapy
**Vaccine Therapy**	NCT01347034	II	20	Radiation Therapy and Intratumoral Autologous Dendritic Cells in Soft Tissue Sarcomas (STS)	External Beam Radiation Therapy
					Autologous Dendritic Cells
	NCT02496520	I and II	6	Dendritic Cell-based Immunotherapy for Advanced Solid Tumours of Children and Young Adults	Dendritic Cells
					Surgery, chemotherapy, and radiation therapy as needed by the patient’s tumor and stage
	NCT00365872	II	17	External Beam Radiation With Intratumoral Injection of Dendritic Cells As Neo-Adjuvant Treatment for Sarcoma	Dendritic Cell Injections
					Radiation therapy
					Complete Resection
	NCT00948961	I and II	70	A Study of CDX-1401 in Patients With Malignancies Known to Express NY-ESO-1	CDX-1401
					Resiquimod (TLR7/8 agonist)
					Hiltonol® (Poly-ICLC, TLR3 agonist)
	NCT03357315	I and II	30	Mix Vaccine for Metastatic Sarcoma Patients	Mix vaccine
	NCT00005628	II	35	Vaccine Therapy in Treating Patients With Recurrent Soft Tissue Sarcoma	Vitespen
	NCT00001564	II	30	A Pilot Study of Tumor-Specific Peptide Vaccination and IL-2 With or Without Autologous T Cell Transplantation in Recurrent Pediatric Sarcomas	EF-1, EF-2, PXFK, and E7 peptides
					IL-2, IL-4, GM-CSF, and CD40 Ligand
	NCT00003408	II	40	Biological Therapy Following Chemotherapy and Peripheral Stem Cell Transplantation in Treating Patients With Cancer	Aldesleukin (synthetic IL-2) Recombinant interferon alfa Sargramostim (recombinant GM-CSF)
	NCT00923351	I and II	44	Therapy to Treat Ewing’s Sarcoma, Rhabdomyosarcoma or Neuroblastoma	Tumor Purged/CD25 Depleted Lymphocytes
					Tumor Purged/CD25 Depleted Lymphocytes with Tumor Lysate/KLH Pulsed Dendritic Cell Vaccine
					rhIL-7
					Tumor Lysate/KLH Pulsed Dendritic Cell Vaccine
	NCT02423863	II	26	In Situ, Autologous Therapeutic Vaccination Against Solid Cancers With Intratumoral Hiltonol®	Hiltonol® (Poly-ICLC, TLR3 agonist)

## Data Availability

Not applicable.
